# Thermal and Flow Analysis of Fully Developed Electroosmotic Flow in Parallel-Plate Micro- and Nanochannels with Surface Charge-Dependent Slip

**DOI:** 10.3390/mi13122166

**Published:** 2022-12-08

**Authors:** Long Chang, Yanjun Sun, Mandula Buren, Yongjun Jian

**Affiliations:** 1 School of Mathematical Science, Inner Mongolia University, Hohhot 010021, China; 2 School of Statistics and Mathematics, Inner Mongolia University of Finance and Economics, Hohhot 010070, China; 3 School of Mathematical Science, Inner Mongolia Normal University, Hohhot 010022, China

**Keywords:** electroosmotic flow, velocity slip, surface charge-dependent, high zeta potential, fully developed, joule heating

## Abstract

This study analytically investigates the coupled effects of surface charge and boundary slip on the fully developed electroosmotic flow and thermal transfer in parallel plate micro and nanochannels under the high zeta potential. The electric potential, velocity, temperature, flow rate, and Nusselt number are obtained analytically. The main results are that the velocity of bulk flow is significantly reduced in the presence of the surface charge-dependent slip. Moreover, the maximum velocity at *ζ* = −125 mV is approximately twice as large as that at *ζ* = −25 mV. The velocity and dimensionless temperature increase as the zeta potential increases. The dimensionless temperature of the surface charge-dependent slip flow is larger than that of the surface charge-independent slip flow. For the surface charge-dependent slip flow, the maximum temperature at *ζ* = −125 mV is approximately four times larger than that at *ζ* = −25 mV. The Nusselt number decreases with Joule heating and increases with a positive heat transfer coefficient. The Nusselt number decreases as the electric field and the magnitude of the zeta potential increase. In the surface charge-dependent slip flows, the Nusselt number is smaller than that in the surface charge-independent slip flows.

## 1. Introduction

Over the last decades, the micro- and nanoscale fluid systems based on physics, medicine, chemistry and biology have been widely applied in various fields [1,2], such as the separation and detection of chemical and biological samples, the design of heat and mass transfer system, micro-and nanoflow control in lab-on-chip [2]. In these applications, the thermal properties of fluid flows are significant factors affecting the performance of micro-nanoscale fluid systems, including the temperature of fluid could affect the properties of biological and medical samples [3], as well as the formation and stability of nanobubbles at the solid–liquid interface [4]. Traditionally, compared with macroscopic flows, the reduced characteristic scale of microfluidic channels results in surface forces that are much larger than the volume force. Therefore, for certain aspects like surface force, capillary effect, slip effect, and rapid heat transfer effect [5], the micro- and nanofluidic mechanics exhibit new characteristics distinguished from the macro-scale fluid flow. At present, several mechanisms for fluid-driving in micro- and nanoflows have been put forward in the existing literature, such as the micropumps by pressure gradient [6], external electric field, external magnetic field [7,8,9,10], surface tension, and acoustic wave [11]. In most cases, a solid surface acquires a negative electric charge when brought into contact with an electrolyte solution. In return, the charged solid surface redistributes nearby ions in the surrounding solution forming a double-layer structure of ions in the vicinity of the surface of the solid, which is referred to as the electric double layer (EDL) [2,12]. The EDL is a region close to the charged surface, where the counter ions exceed the ions, thus neutralizing the surface charge. When an axial electric field is applied, the electric power generated by the interaction between the net charge density in EDL and the applied electric field can drive the flow. This kind of flow is known as electroosmotic flow (EOF) [2,12].

Due to its relative advantages, such as simple design requirements and no need for moving elements, the electroosmosis force has been extensively discussed as a more effective driving mechanism than a simple pressure driving mechanism. In this respect, much of the literature on the microchannel electroosmosis flow by Newtonian fluid and non-Newtonian fluid has been well studied [13,14,15,16,17,18,19,20,21,22,23,24,25]. Yang et al. [17] analyzed the EOF in an annulus with high zeta potentials. Kadet and Koryuzlov [22] have presented the results of experimental studies of EOF of two immiscible liquids in a thin slit channel. Through experimental, simulation and theoretical analyses in polymer-coated slits, Monteferrante and Melchionna [23] pointed out that electroosmotic suppression arises from the frictional forces acting on the ionic currents.

However, when the voltage is high, Joule heating is inevitable in the process of EOF. How to dramatically improve the performance of conventional heat transfer has become a major challenge for scientists and engineers. Therefore, heat transfer has become the focus of EOF research in recent years [26,27,28,29,30,31,32,33,34,35,36,37,38]. Under the condition of a constant heat flux boundary, Maynes and Webb [31] studied the thermal transport of EOF in parallel plates and circular microchannels and obtained the analytical expression of temperature distribution. In addition, they also studied the viscous dissipation effect [32]. Liechty et al. [33] investigated the fully-developed convection heat transfer of EOF in a circular microtube with arbitrary wall zeta potentials under imposed wall temperatures and imposed wall heat fluxes. Tang et al. [34] have obtained a numerically transient temperature field that takes into account Joule heating in a microchannel. Chakraborty [35] has gained analytical solutions of the temperature and the Nusselt number of the fully developed flow driven by both electroosmotic force and imposed pressure gradient. Sadeghi and Saidi [36] have investigated the influence of viscous dissipation on thermal transport characteristics of the fully developed combined electroosmotic and pressure-driven flow in a parallel-plate microchannel with uniform wall heat flux. The research results show that viscous dissipation and Joule heating dominate the heat transfer problem in EOF.

However, most studies on EOF problems are limited to no-slip boundary conditions. Actually, the experimental results show that when the fluid flows over the solid surface, there may be relative motion between the solid surface and the liquid near the solid surface. The velocity of the solid surface is called slip velocity *u_slip_*, which is related to slip length *b*. The slip length is usually in the range of several nanometers to tens of micrometers [39]. Previous studies have shown that EDL and slip can significantly affect micro- and nanoscale fluid behaviors [3,40,41,42,43,44,45,46,47,48,49]. In the case of electroosmotic flow, Rojas et al. [19] conducted a dispersion analysis in the neutral non-reacting solute in the circular microchannel with boundary slip and concluded that the existence of slip can greatly magnify the dispersion increase caused by the induced pressure gradient caused by the non-uniform wall potential [20]. Kundu and Saha [24] discussed the thermal transport behavior of electrostatic-driven flow in microchannels based on the no-slip, first- and second-order slip at the boundary of velocity distribution and the no-jump, first- and second-order heat slip of thermal response under the condition of maintaining uniform wall heat flux. In a microchannel between two parallel plates with imposed heat flux, the liquid flows with the slip boundary condition have been numerically investigated by Ngoma and Erchiqui [38]. Park and Kim [46] studied the Electrokinetic slip flow through hydrophobic microchannels and obtained an analytical solution of volumetric flow rate and solute retention time.

Although most of the studies in the literature were made to investigate the effects of EOF and convective heat transfer on liquid flow, the impacts of the surface charge about boundary slip on heat transfer in electroosmosis flows were rarely concerned about [40,50]. Under the influence of the surface charge, EDL with an opposite net charge is formed in the solution near the surface of the wall. Then, this charge distribution near the wall results in an attractive electric force between the charged wall and the nearby fluid. Eventually, this interaction between the wall and the fluid further influences the slip boundary conditions [41,50]. Utilizing the molecular dynamics simulation method, Joly et al. [50] studied and used a mathematical model to describe the effect of surface charge on the boundary slip. Jing and Bhushan [41,51] used atomic force microscopy to study the relationship between the boundary slip and the surface charge and found the same relationship as that obtained by Joly et al. [50]. So far, although most researchers have found the dependence of boundary slip on a surface charge, the study of fluid types is mainly limited to Newtonian fluid [50,51,52,53,54,55,56]. Bhushan et al. [40,41] studied the effect of surface charge-dependent boundary slip on the electroviscous effect between microchannels. Pan et al. [57,58] further investigated the effect of surface charge-dependent slip on the electroviscous effect and heat transfer in a microchannel and obtained analytical expressions for velocity, temperature distributions, and Nusslt number.

The EOFs in slit channels have a variety of practical applications, such as micro/nano-pumping, mixing, and oil extraction [22]. However, a large number of previous studies rarely discussed this problem theoretically. The purpose of this paper is to investigate the effects of the surface charge-dependent slip on the combined electroosmotic and pressure-driven flow in a parallel-plate micro and nanochannel with high zeta potential and constant wall heat flux. The paper is organized as follows: Section 2 presents the mathematical modeling. Results and comparisons are discussed in Section 3. The conclusions are drawn in Section 4.

## 2. Mathematical Modeling

### 2.1. Electric Potential Distribution

**Figure 1:**
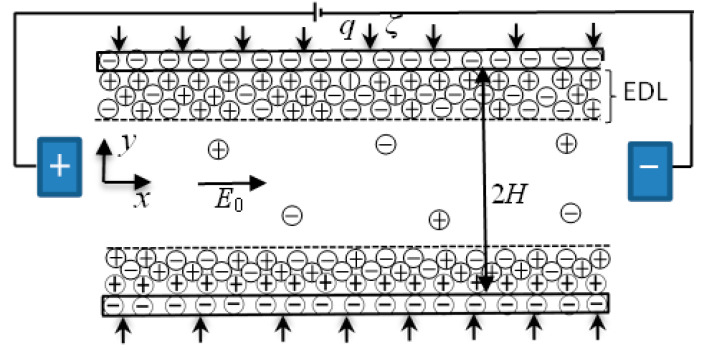
Schematic of electroosmotic flow between two parallel plates with surface charge-dependent slip and constant heat flux.

In this study, the steady combined electroosmotic and pressure-driven flow of a symmetric binary electrolyte between two parallel plates with surface charge-dependent slip and high zeta potential is studied with density *ρ*, dynamic viscosity *μ*, and electric conductivity *σ* of the fluid. A Cartesian orthonormal coordinate system (*x*, *y*, *z*) is used with the origin fixed at the middle of the channel, as shown in Figure 1. The channel width *W* in the *z*-direction and length *L* in the *x*-direction is much larger than the channel height 2*H* in the *y*-direction, i.e., 2*H << L* and *W << L*. EOF in the *x*-direction is generated when an electric field is applied along the *x*-direction. On the boundary, the effect of surface charge density *σ_s_* on the boundary slip *b* is considered [41,53]:

(1) $$b=\frac{b_{0}}{1+\left(1/\alpha \right){\left(\sigma_{s}d^{2}\;/e\right)}^{2}\left(l_{B}/d^{2}\right)b_{0}},$$

where *b*_0_ is the original slip length without considering surface charge effect, α~1 is a numerical factor, *e* is the elementary charge, *d* is the equilibrium distance of Lennard-Jones potential, and *l_B_* = *e*^2^/(4*πεk_B_T_av_*), *ε* is the permittivity of the electrolyte solution, *k_B_* is the Boltzmann constant and *T_av_* is the average absolute temperature over the channel cross-section [53,54]. According to the EDL theory, the electric potential ψ and the net charge density *ρ_e_* satisfy Poisson equation [2,12]

(2) $$\nabla^{2}\psi =-\frac{\rho_{e}}{\varepsilon }.$$

The ideal solution of fully dissociated symmetric salt, the electric charge density is given by [2,12]:

(3) $$\rho_{e}=-2n_{0}z_{v}e\sinh\frac{z_{v}e\psi }{k_{B}T_{av}},$$

where *n*_0_ is the ion density, *z_v_* is the valence number of ions in solution. Using Equations (2) and (3), we can obtain the Poisson–Boltzmann equation

(4) $$\frac{d^{2}\psi }{dy^{2}}=\frac{2n_{0}z_{v}e}{\varepsilon }\sinh\left(\frac{z_{v}e\psi }{k_{B}T_{av}}\right)\;.$$

The following dimensionless parameters are introduced:

(5) $$\bar{\psi }=\frac{z_{v}e\psi }{k_{B}T_{av}}\;,\;\bar{y}=\frac{y}{H}\;,\;K=\kappa H,$$

where *K* is called the nondimensional electrokinetic width, *κ =* (2*n*_0_*z_v_*^2^*e*^2^*/*(*εk_B_T_av_*))^1/2^ is Debye–Hückel parameter and 1/*κ* is the characteristic thickness of the EDL. The dimensionless form of the Poisson–Boltzmann equation can be expressed as:

(6) $$\frac{d^{2}\bar{\psi }}{d{\bar{y}}^{2}}=K^{2}\sinh\bar{\psi }.$$

Equation (6) is subject to the following boundary conditions:

(7) $${\bar{\psi }|}_{\bar{y}=-1}=Z,\;\;\frac{d\bar{\psi }}{d\bar{y}}{|}_{\bar{y}=0}=0,$$

here, *Z* is the dimensionless zeta potential, i.e., *Z* = *z_v_eζ*/(*k_B_T_av_*).

From Equation (6) and boundary conditions (7), one obtains the dimensionless potential distribution:

(8) $$\bar{\psi }=4\tanh^{-1}\left[e^{-K\left(1+\bar{y}\right)}\tanh\left(Z/4\right)\right].$$

### 2.2. Velocity Distribution

Since the velocity of EOF and the hydraulic diameter of micro/nano channels are very small, the Reynolds number is very low, and hence fully-developed laminar flow can be assumed. In the fully-developed parallel flow $(\frac{\partial u}{\partial x}=0,v=0,w=0$), the Navier–Stokes equation becomes linear. Here, *u, v,* and *w* are the velocities in the *x, y,* and *z*-directions, respectively. The momentum equation in the *x*-direction under the combined action of pressure and electric field *E*_0_ is as follows:

(9) $$\mu \frac{d^{2}u}{dy^{2}}=\frac{dp}{dx}-\rho_{e}E_{0}.$$

Equation (9) is subject to the following boundary conditions:

(10) $$u{|}_{y=-H}=b\frac{du}{dy}{|}_{y=-H},\;\;\;\frac{du}{dy}{|}_{y=0}=0,$$

where the slip length *b* is given by Equation (1).

According to the charge conservation law, the surface charge should be equal to the net charge in the fluid. Therefore

(11) $$\sigma_{s}=-\int_{-H}^{0}\rho_{e}dy=-\varepsilon \frac{d\psi }{dy}{|}_{y=-H}.$$

To write the governing equations and the boundary conditions in dimensionless form, let:

(12) $$\bar{u}=\frac{u}{U_{eo}},\;U_{eo}=-\frac{\varepsilon \zeta E_{0}}{\mu },\bar{\;b}=\frac{b}{H},$$

where *U_eo_* is the steady Helmholtz–Smoluchowski velocity. First, substituting Equation (12) in the governing equations and in their boundary conditions and then solving the corresponding dimensionless boundary value problem by integrating Equation (9) twice, we obtain

(13) $$\bar{u}=u_{r}\left(1+2\bar{b}-{\bar{y}}^{2}\right)+\left[Z+2\bar{b}K\sinh\left(Z/2\right)-\bar{\psi }\right]/Z,$$

in which *u_r_* = *u_max_*/*U_eo_* is the ratio of the velocity of pressure-driven flow and that of EOF between two parallel plates, *u_max_ = −H*^2^ (2*μ*)^−1^ d*p*/d*x*. Note that *u_r_* = 0 corresponds to a pure EOF.

According to the definition of the dimensional volumetric flow rate, we have

(14) $$Q^{*}=2\int_{0}^{W}dz\int_{-H}^{0}udy==2WU_{eo}H\int_{-1}^{0}\bar{u}d\bar{y}.$$

Introduce a dimensionless volumetric flow rate

(15) $$Q=\frac{Q^{*}}{WU_{eo}H}.$$

Substituting Equation (13) into Equation (14), the dimensionless volumetric flow rate can be expressed as

(16) $$Q=4u_{r}\left(\frac{1}{3}+\bar{b}\right)+2+\frac{4\bar{b}K}{Z}\sinh(\frac{Z}{2})-\frac{2}{Z}\int_{-1}^{0}\bar{\psi }d\bar{y}.$$

The integral in Equation (16) can be calculated numerically using Gaussian quadrature.

### 2.3. Temperature Distribution

Energy conservation, including the effects of Joule heating and viscous dissipation, can be written as

(17) $$\rho c_{p}u\frac{\partial T}{\partial x}=k\left(\frac{\partial^{2}T}{\partial x^{2}}+\frac{\partial^{2}T}{\partial y^{2}}\right)+s+\mu {\left(\frac{du}{dy}\right)}^{2},$$

where *s* and *μ*(d*u*/d*y*)^2^ denote the rate of volumetric heat generation due to Joule heating and viscous dissipation, respectively. *s* = *i_e_*^2^*σ_ρ_*, where *i_e_* is the current density and *σ_ρ_* is the liquid electrical resistivity. The current density is given by [33,36]

(18) $$i_{e}=\frac{E_{0}}{\sigma_{\rho }}\cosh\bar{\psi },$$

where *σ_ρ_*^−1^ = 2*z_v_*^2^*e*^2^*Dn*_0_/(*k_B_T_av_*), *D* is the diffusivity of ions in the electrolyte. The boundary conditions for the energy equation are as follows:

(19) $$\frac{\partial T}{\partial y}{|}_{y=0}=0,k\frac{\partial T}{\partial y}{|}_{y=-H}=-q\;or\;T{|}_{y=-H}=T_{w}\left(x\right).$$

The dimensionless temperature *θ* is introduced

(20) $$\theta \left(y\right)=\frac{T-T_{w}}{qH/k}.$$

This depends only on *y* for the fully developed flow. Differentiating both sides of Equation (20) with respect to x gives

(21) $$\frac{\partial T}{\partial x}=\frac{dT_{w}}{dx}=\frac{dT_{b}}{dx}=const,$$

in which *T_b_* is the bulk temperature. Considering an energy balance on a control volume with thickness d*x* along the centerline of the channel, we can write Equation (17) as:

(22) $$\rho c_{p}\frac{\partial T_{b}}{\partial x}\int_{-H}^{0}udy=q+\frac{E_{0}^{2}}{\sigma_{\rho }}\int_{-H}^{0}\cosh^{2}\bar{\psi }dy+\mu \int_{-H}^{0}{\left(\frac{du}{dy}\right)}^{2}dy.$$

From Equation (22), we have

(23) $$\frac{dT_{b}}{dx}=\frac{1}{\rho c_{p}u_{m}H}\left(q+\frac{HE_{0}^{2}}{\sigma_{\rho }}\beta +\frac{\mu U_{eo}^{2}}{H}\gamma \right),$$

where *u_m_*, *β*, *γ* are given by

(24) $$u_{m}=U_{eo}\int_{-1}^{0}\bar{u}d\bar{y},\beta =\int_{-1}^{0}\cosh^{2}\bar{\psi }d\bar{y},\;\gamma =\int_{-1}^{0}{\left(\frac{d\bar{u}}{d\bar{y}}\right)}^{2}d\bar{y}.$$

So the energy equation in dimensionless form can be written as

(25) $$\frac{d^{2}\theta }{d{\bar{y}}^{2}}=\frac{1+S\beta +Br\gamma }{{\bar{u}}_{m}}\bar{u}-S\cosh^{2}\bar{\psi }-4Br{\left(u_{r}\bar{y}-\frac{K}{Z}\sinh\frac{\bar{\psi }}{2}\right)}^{2},$$

where ${\bar{u}}_{m}$ = *u_m_*/*U_eo_* is the dimensionless axial mean velocity, *Br* = *μU*^2^*_eo_*/*qH* is the Brinkman number, and *S* = *E*_0_^2^*H*/*σ_ρ_q* is the dimensionless volumetric heat generation due to Joule heating. The thermal boundary conditions in the dimensionless form can be written as

(26) $$\frac{d\theta }{d\bar{y}}{|}_{\bar{y}=0}=0,\;\theta {|}_{\bar{y}=-1}=0.$$

Integrating Equation (25) twice and using the boundary conditions (26), we obtain

(27) $$\theta =f\left(\bar{y}\right)-f\left(-1\right),$$

here

(28) $$\begin{array}{cc} f\left(\bar{y}\right)= & \frac{1+S\beta +Br\gamma }{2{\bar{u}}_{m}}\{u_{r}\left[\left(1+2\bar{b}\right){\bar{y}}^{2}-\frac{{\bar{y}}^{4}}{6}\right]-\frac{2}{Z}\int_{0}^{\bar{y}}d\tilde{y}\int_{0}^{\tilde{y}}\hat{\psi }d\hat{y}+\left[Z+2\bar{b}K\sinh\left(\frac{Z}{2}\right)\right]\\ & -S\int_{0}^{\bar{y}}d\tilde{y}\int_{0}^{\tilde{y}}\cosh^{2}\hat{\psi }d\hat{y}-4Br\int_{0}^{\bar{y}}d\tilde{y}\int_{0}^{\tilde{y}}{\left[u_{r}\hat{y}-\frac{K}{Z}\sinh\frac{\hat{\psi }}{2}\right]}^{2}d\hat{y}. \end{array}$$

In the above equation, for simplicity, $\hat{\psi }=\bar{\psi }\left(\hat{y}\right)$. To obtain the Nusselt number, the dimensionless bulk temperature *θ_b_* must be calculated first, which is given by

(29) $$\theta_{b}=\frac{\int_{-1}^{0}\bar{u}\theta d\bar{y}}{\int_{-1}^{0}\bar{u}\;d\bar{y}}=\frac{\int_{-1}^{0}\bar{u}\theta d\bar{y}}{{\bar{u}}_{m}},$$

and Nusselt number is defined as

(30) $$Nu=\frac{2hH}{k}=\frac{2qH}{k\left(T_{w}-T_{b}\right)}=-\frac{2}{\theta_{b}}.$$

## 3. Results and Discussion

In this section, the discrete numerical solutions of flow rate *Q*, dimensionless temperature *θ* and Nusselt number are obtained by Gaussian integration in two parallel plates with surface charge-dependent slip, and constant heat flux, together with the analytical and semi-analytic solutions for velocity, flow rate, and temperature. In addition, a series of results are displayed in the form of graphs to intuitively express the coupling effect of surface charge and slip on the combined pressure and electroosmotic-driven flow in a parallel-plate micro- and nanochannel. In order to obtain veritable and effective results, the following typical parametric values are used [2,12,53,54]: *d* = 0.4 × 10^−9^ m, *α* = 1, *µ* = 1.01 × 10^−3^ Pa∙s, *ρ* = 10^3^ kgm^−3^, *e* = 1.6 × 10^−19^ C, *z* = 1, *T*_av_ = 298 K, *ε* = 7 × 10^−10^ Fm^−1^, *k_B_* = 1.38 × 10^−23^ JK^−1^, *D* = 1.612 × 10^−9^ m^2^s^−1^, without special instructions d*p*/d*x* = 0 Pa/m.

**Figure 2:**
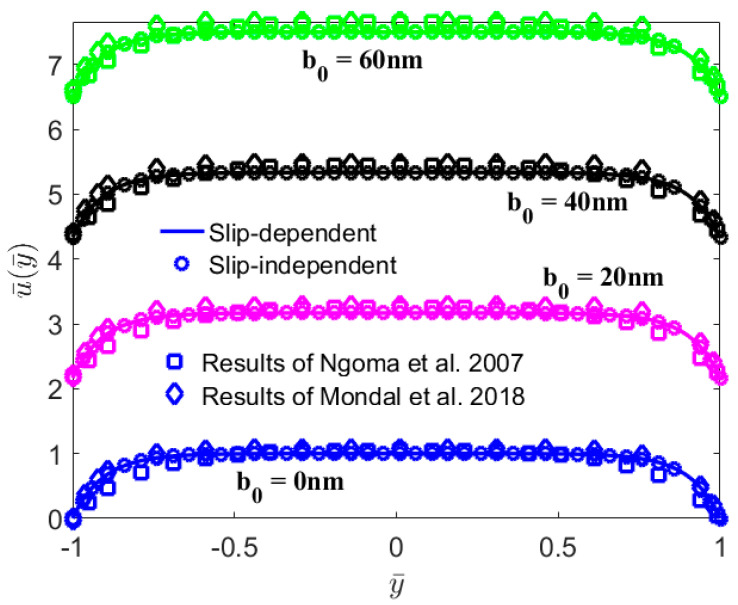
Comparison of slip velocity Equation (13) with those obtained by Mondal et al. [37] and Ngoma et al. [38] (*n*_0_ = 1 mM, *H* = 100 nm, *E*_0_ = 10^6^ V/m, *ζ* = −25 mV).

In Figure 2, the present results in the case of charge-independent slip are compared with those obtained by Mondal et al. [37] and Ngoma et al. [38]. Ngoma and Erchiqui [38] investigated the fluid flow with the slip boundary condition in a microchannel between two parallel plates; that is, the case of low zeta potential and the slip length *b* = *b*_0_. In addition, Mondal and Shit [37] took into account the non-uniform walls varying sinusoidally, which can be simplified into the model in this paper in the limit case. The present results are found to be in agreement with those in Refs. [37,38]. Moreover, there is a slight error near the wall, which is mainly caused by the Debye–Hückel approximation used in Refs. [37,38].

**Figure 3:**
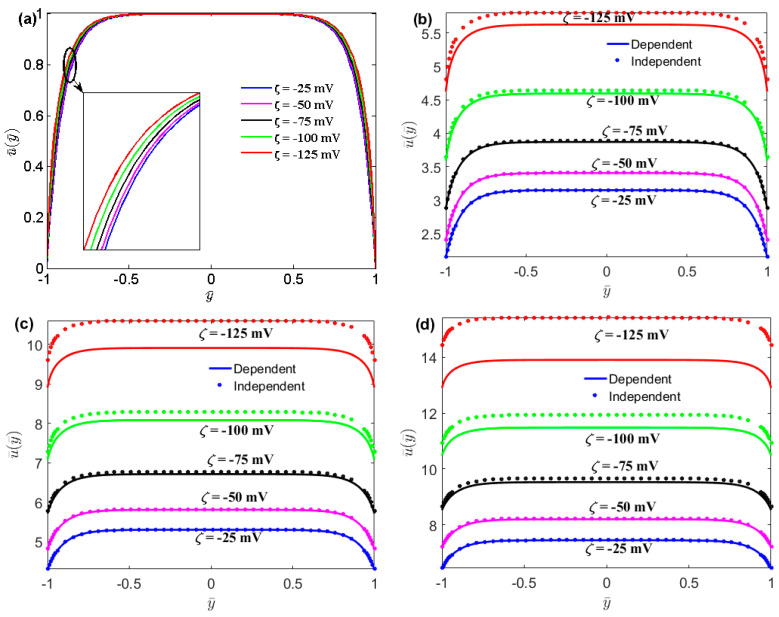
The coupled effects of the zeta potential and the boundary slip on the velocity distribution. (*n*_0_ = 1 mM, *H* = 100 nm, *E*_0_ = 10^6^ V/m);(**a**) *b*_0_ = 0 nm;(**b**) *b*_0_ = 20 nm;(**c**) *b*_0_ = 40 nm;(**d**) *b*_0_ = 60 nm.

In Figure 3, the non-slip velocity profile is plotted as a function of the distance for different values of the zeta potential and slip length. It can be seen from Figure 3a that for large zeta potentials, the velocity distribution of no-slip flow varies with zeta potential in the thin layer near the solid walls. As shown in Figure 3, it is found that the velocities of both the no-slip and slip flows increase with the zeta potential. For the surface charge-dependent slip flow, the maximum velocity at *ζ* = −125 mV is approximately twice as large as that at *ζ* = −25 mV. The study also demonstrated that the velocity of the surface charge-independent slip flow is larger than that of the surface charge-dependent flow. The surface charge-dependent expresses the case of *b* = *b*_0_. In addition, the difference between the velocities of the surface charge-dependent and charge-independent slip flows increases with zeta potential, owing to the slip length decreasing with zeta potential, as shown in Equation (1).

**Figure 4:**
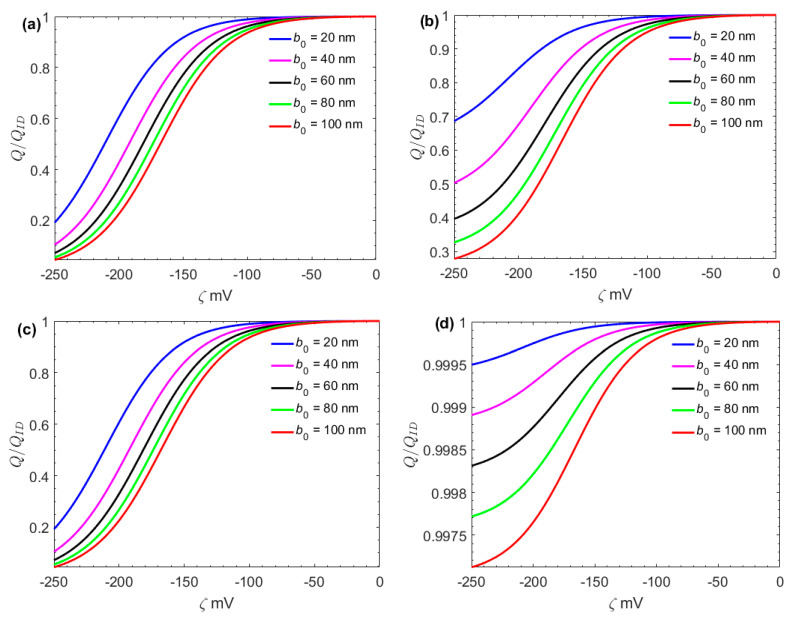
Variation of the ratio *Q/Q_ID_* with *ζ* at different *b*_0_ (*E*_0_ = 10^6^ V/m), where *Q_ID_* is the flow rate without considering the effect of the surface charge on the slip length. (*n*_0_ = 1 mM, *E*_0_ = 10^6^ V/m) (**a**) *H* = 100 nm, dp/dx = 0 kPa/m; (**b**) *H* = 100 nm, dp/dx = 10^3^ kPa/m; (**c**) *H* = 100μm, dp/dx = 0 kPa/m; (**d**) *H* = 100 μm, dp/dx = 10^3^ kPa/m.

In Figure 4, the effect of the surface charge-dependent slip on the flow rate is investigated. Here, the *Q_ID_* represents the flow rate without considering the effect of the surface charges on the slip length. The surface charge effect is increased when the magnitude of zeta potential *ζ* increases and the slip length *b_0_* increases. Obviously, the slip length decreases with the zeta potential. A reasonable explanation is the increase of the attractive electrostatic force between the charged solid surface and the liquid near the solid–liquid interface. In addition, Figure 4 shows that the surface charge effect on the slip length in the microchannel is smaller than in the nanochannel.

**Figure 5:**
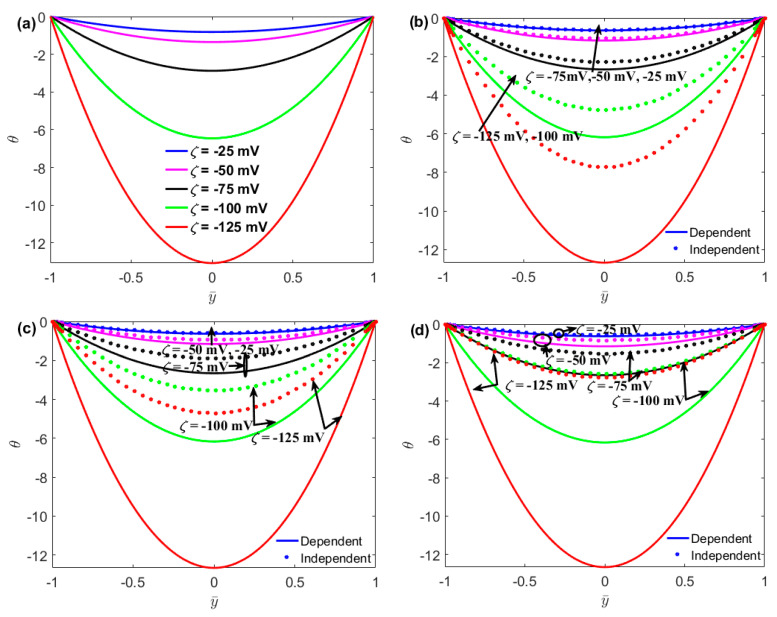
The coupling effects of the zeta potential and the boundary slip on the temperature distribution. (*n*_0_ = 10 mM, *H* = 100 nm, dp/dx = 800 kPa/m, *E*_0_ = 10^6^ V/m, *q* = 10^3^ W/m^2^);(**a**) *b*_0_ = 0 nm;(**b**) *b*_0_ = 20 nm;(**c**) *b*_0_ = 40 nm;(**d**) *b*_0_ = 60 nm.

In Figure 5, the temperature profile is plotted against the distance for different values of the zeta potential and the slip length. As seen in Figure 5, the dimensionless temperature in the nanochannel increases when the magnitude of the zeta potential increases. The reason is that as the magnitude of the zeta potential increases, the velocity and hence the effective Joule heating increases, and thus the heat transfer can be enhanced. Furthermore, it can be seen from Figure 5 that the dimensionless temperature in the surface charge-dependent slip flow is larger than that in the surface charge-independent slip flow. The viscous dissipation behaves like an energy source, increasing the temperature of the fluid, especially near the wall. The reason is that the highest shear rate occurs at this region while it is zero at the centerline. In addition, since the walls are cooling, heat is transferred from the interior to the exterior of the fluid, and fluid convection carries away a fraction of this heat, so the amplitude of the fluid velocity is large, further leading to a decrease in the amplitude of the temperature. For the surface charge-dependent slip flow, the maximum temperature at *ζ* = −125 mV is approximately four times larger than that at *ζ* = −25 mV.

The total Joule heating per unit length is defined as

(31) $$E_{JH}=\int_{-H}^{H}i_{e}^{2}\sigma_{\rho }dy.$$

Nondimensionalizing this parameter by the total Joule heating per unit length in the case of low zeta potential, 2*H* (*E*_0_^2^/*σ_ρ_*), we obtain

(32) $$E_{e}=\frac{E_{JH}}{2HE_{0}^{2}/\sigma_{\rho }}=\int_{-1}^{0}\cosh^{2}\bar{\psi }d\bar{y}=\beta .$$

**Figure 6:**
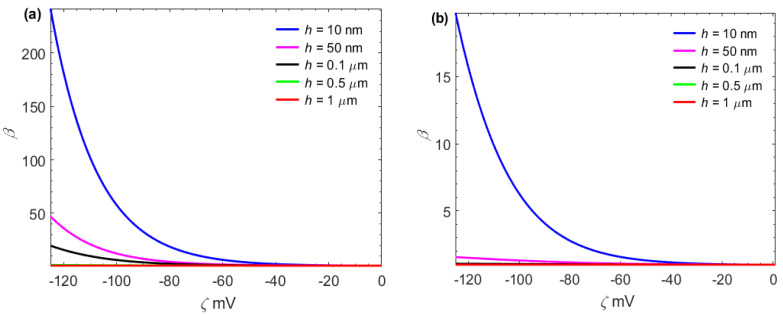
Normalized Joule heating per unit length of the microchannel as a function of *ζ* and *H*.(**a**) *n*_0_ = 1 mM;(**b**) *n*_0_ = 100 mM.

The dimensionless total Joule heating per unit length of the channel *E_e_* is plotted as a function of zeta potential *ζ* in Figure 6 for different values of *H* and *n*_0_. It is found that for low zeta potential (*ζ* < 25 mV), *E_e_* = 1 at all values of *H*. The reason can be illustrated that for low zeta potential, cosh $\bar{\psi }$~1. For a given zeta potential, the effective Joule heating *E_e_* decreases as the half-height *H* of the micro and nanochannel increases. On the contrary, for a given *H*, the zeta potential strengths the effective Joule heating *E_e_*, especially in nanochannels. By comparing Figure 6a,b, it can be found that ion density *n*_0_ can inhibit the increase of Joule heat.

For a given *H*, as the zeta potential *ζ* increases, the effective Joule heating *E_e_* increases, especially in nanochannels. In addition, as the ion density *n*_0_ increases, the effective Joule heat *E_e_* decreases. The reason is that an increase in the channel height *H,* or in the ion density *n*_0_, implies an increase in the dimensionless electrokinetic width *K;* that is, the EDL becomes thinner.

**Figure 7:**
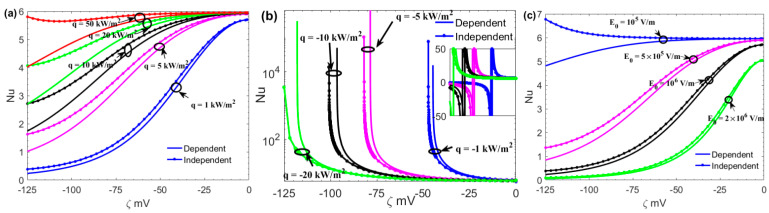
Variations of Nusselt number with *ζ* at different values of *q* and *E*_0_. (*n*_0_ = 10 mM, dp/dx = 800 kPa/m, *H* = 100 nm, *b*_0_ = 20 nm). (**a**) *E*_0_ = 10^6^ V/m; (**b**) *E*_0_ = 10^6^ V/m; (**c**) *q* = 1 kW/m^2^.

Figure 7 depicts the variations of the Nusselt number with zeta potential *ζ* for different values of the surface heat flux *q* and the electric field *E*_0_. Both positive and negative values of the wall heat flux are considered, where positive and negative values of *q* correspond to the case where the fluid is cooled and heated, respectively. In general, increasing the *Br* decreases the Nusselt number, so the *Br* decreases with the heat flow flux *q*. It can be seen that the Brinkman number has a greater influence on the Nusselt number at greater values of heat flux *q* (see Figure 7a). From Figure 7, we can see that the Nusselt number increases with the positive *q* because the convective heat transfer coefficient *h* increases with *q*. For a given constant wall heat flux *q*, the Nusselt number is large when *E*_0_ and *ζ* are small. In contrast to the wall-heating case, in the presence of viscous heating, which occurs at large velocity gradients, *q* has a greater effect on the Nusselt number. As a result of viscous heating, the bulk temperature is not affected significantly, but the wall temperature is greatly increased, and the Nusselt number will be much higher. Due to the large value of viscous heating, the wall temperature is much higher than the bulk temperature. This behavior of the Nusselt number is accompanied by the occurrence of a singularity in Nusselt number values of the wall heating cases; as a result of changing the sign of dimensionless bulk temperature from negative to positive values (see Figure 7b). Increasing the value of *E*_0_ leads to smaller values of the Nu, indicating that wall temperatures increase rather than bulk temperatures when Joule heating is applied. The reason is that although the distribution of energy generated by Joule heating is uniform throughout the channel cross-section, the energy transferred by convection decreases near the wall, and it equals zero at the wall (see Figure 7c). Moreover, Nu is smaller in the surface charge-dependent slip flow than in the surface charge-independent slip flow. The reason is that the convective heat transfer coefficient *h* is reduced by the decrease of the velocity on the boundary due to the surface charge effect on the slip.

## 4. Conclusions

In the present study, the fully developed combined pressure and electroosmotic driven flows in parallel plate micro and nanochannels with surface charge-dependent slip and high zeta potentials have been studied. The classical boundary conditions of surface charge-dependent slip and uniform wall heat flux are taken into account in the analysis. The effects of Joule heating and viscous dissipation are also considered. The electric potential in EDL, velocity, temperature, and Nusselt number have also been obtained analytically. The problem studied here is found to be governed by five parameters: the ratio of the velocity of pressure-driven flow and the velocity of EOF, the electrokinetic width (Debye–Hückel parameter), the zeta potential, Joule heating term, and Brinkman number. The main results of this study can be summarized as follows:

- For a given large zeta potential, the EDL velocity variation is limited to the thin layer that clings to both solid walls.
- Compared with the velocity of the surface charge-independent slip flow, that of the surface charge-dependent slip flow showed a significant reduction.
- The increase in zeta potential causes an increase in the dimensionless temperature in the nanochannel.
- The dimensionless temperature of the surface charge-dependent slip flow is larger than that of the surface charge-independent slip flow.
- For a given zeta potential, the effective Joule heating *E_e_* decreases as the channel height increases; for a given channel height, as the zeta potential *ζ* increases, the effective Joule heating *E_e_* is increased, especially in the nanochannel.
- Ion density *n*_0_ can inhibit the increase of Joule heat.
- The Nu decreases with Joule heating *S* and increases with positive heat transfer coefficient *q*.
- The Nu is large when the applied electric field *E*_0_ and zeta potential *ζ* are small.
- The Nu in the surface charge-dependent slip flow is smaller than that in the surface charge-independent slip flow.

## Data Availability

Not applicable.

## Nomenclature

(*x*, *y*, *z*)	Cartesian orthonormal coordinate system
*W*	channel width
*H*	half channel height
*L*	channel length
*b*	slip length
*b*_0_	original slip length without considering the surface charge effect
*e*	elementary charge
*d*	equilibrium distance of Lennard-Jones potential
*T_av_*	average absolute temperature
*k_B_*	Boltzmann constant
*n*_0_	ion density of bulk liquid
*z_v_*	valence of ion
*K*	dimensionless electrokinetic width *= κH*
$\bar{y}$	dimensionless Cartesian coordinate system
*E_0_*	electric field strength
Z	dimensionless zeta potential
*u*	axial velocity
*P*	pressure
*U_eo_*	Helmholtz−Smoluchowski electroosmotic velocity = *–εζE*_0_/*μ*
$\bar{u}$	dimensionless axial velocity
$\bar{b}$	dimensionless slip length
*u_r_*	ratio of the velocity of pressure-driven flow and that of EOF between two parallel plates = *u_max_*/*U_eo_*
*u_max_*	velocity of pressure-driven flow = *−H*^2^ (2*μ*)^−1^ d*p*/d*x*
*Q**	volumetric flow rate
*Q*	dimensional volumetric flow rate
*Q_ID_*	flow rate without considering the effect of the surface charge on the slip length
*s*	rate of volumetric heat generation due to Joule heating = *i_e_*^2^*σ_ρ_*
*D*	the diffusivity of ions in the electrolyte
*c_p_*	specific heat at constant pressure
*k*	thermal conductivity
*T*	temperature
*i*_e_	current density
*T_b_*	bulk temperature
*T_w_*	wall temperature
*u_m_*	axial mean velocity
${\bar{u}}_{m}$	dimensionless axial mean velocity = *u_m_*/*U_eo_*
*Br*	Brinkman number = *μU_eo_*^2^/*qH*
*S*	dimensionless volumetric heat generation due to Joule heating = *E*_0_^2^*H*/*σ_ρ_q*
Nu	Nusselt number
*E_JH_*	total Joule heating per unit length
*E_e_*	dimensionless total Joule heating
*h*	heat transfer coefficient
Greek symbols	
*ρ*	density
*σ*	electric conductivity
*σ_s_*	surface charge density
α	a numerical factor
*ε*	dielectric constant
*ψ*	electric potential
*ζ*	zeta potential
*κ* ^−^^1^	Debye length
*ρ_e_*	net volumetric charge density
$\bar{\psi }$	dimensionless electric potential
*μ*	dynamic viscosity
*σ_ρ_*	liquid electrical resistivity
*θ*	dimensionless temperature
*β*	integral parameter
*γ*	integral parameter
Subscripts	
*av*	average
*b*	bulk
*w*	wall
*m*	mean
*JH*	Joule heating
s	surface
*r*	ratio
